# Age-specific effects on adverse pregnancy outcomes vary by maternal characteristics: a population-based retrospective study in Xiamen, China

**DOI:** 10.1186/s12889-023-15235-4

**Published:** 2023-02-14

**Authors:** Caoxin Huang, Qiuhui Jiang, Weijuan Su, Fuping Lv, Jinyang Zeng, Peiying Huang, Wei Liu, Mingzhu Lin, Xuejun Li, Xiulin Shi, Xuanling Zheng

**Affiliations:** 1 Department of Endocrinology and Diabetes, Xiamen Diabetes Institute, Xiamen Clinical Medical Center for Endocrine and Metabolic Diseases, Xiamen Diabetes Prevention and Treatment Center, Fujian Key Laboratory of Diabetes Translational Medicine, School of Medicine, The First Affiliated Hospital of Xiamen University, Xiamen University, Xiamen, Fujian China; 2 The School of Clinical Medicine, Fujian Medical University, Fuzhou, Fujian China

**Keywords:** Advanced maternal age, Pregnancy outcomes, China

## Abstract

**Background:**

Advanced maternal age (AMA; ≥35 years) is considered to be a major risk factor for adverse pregnancy outcomes. Along with the global trend of delayed childbearing, and in particular, the implementation of China’s second and third-child policy leading to a dramatic increase of AMA in recent years, the association between maternal age and pregnancy outcomes requires more investigation.

**Methods:**

A population-based retrospective study was performed. Data were derived from the Medical Birth Registry of Xiamen from 2011 to 2018. Univariate and multivariate logistic regression was used to evaluate the effects of maternal age on pregnancy outcomes.

**Results:**

A total of 63,137 women categorized into different age groups (< 25 years, 25–29 years, 30–34 years, and ≥ 35 years) were included in this study. Compared with the mothers aged 25–29 years, the univariate regression analysis showed that mothers aged < 25 years had lower risks of gestational diabetes mellitus (GDM) and cesarean. AMA was associated with higher risks of GDM, hypertension, cesarean, preterm birth, low-birth weight (LBW), large-for-gestational-age (LGA), macrosomia, and stillbirth (all P < 0.01). After adjustment for potential confounding factors, increased risks of GDM, hypertension, cesarean, preterm birth, and LBW remained significantly associated with AMA (all P < 0.05), whereas AMA mothers showed a lower risk of macrosomia than their younger counterparts. Additionally, no significant differences were detected in terms of Apgar score < 7.

**Conclusion:**

AMA was associated with adverse pregnancy outcomes including increased risks of GDM, hypertension, cesarean, preterm birth, and LBW. This study confirmed the relationship between AMA and certain adverse maternal and fetal outcomes and emphasizes the necessity for women to be cautious about the age at which they become pregnant.

## Introduction

Advanced maternal age (AMA), defined as pregnancy at the age of 35 years or above, is considered a significant risk factor for adverse pregnancy and perinatal outcomes [1, 2]. Over the past few decades, a remarkable shift in the demographic changes of maternal age has been observed worldwide. Data from the United States [3] showed that pregnant women aged 35 to 39 years have increased by 36% from 1991 to 2001, and the incidence of pregnancy in women aged 40 to 44 years has elevated by 70% significantly in the same period. Surprisingly, pregnant women aged between 50 and 54 years gave birth to 263 infants in 2002 [3]. Nowadays, prolonged education, career priority, financial stress, lack of flexible working hours, and fertility control via effective contraceptive methods have contributed to a postponed age at pregnancy [4–6]. Apparently, the AMA has become a trend attributed to economic and social influences.

According to data published in China Statistical Yearbook in 2015, the birth rate for women in China aged 35 to 39 years has increased from 8.65% to 2004 to 17.04% in 2014, and from 1.77 to 3.96% for those aged 40 to 44 years. In contrast, the birth rate for women aged 25–29 years declined from 102.44 to 93.62% [7]. According to data published in 2016, the rate of late pregnancy accounted for approximately 31% of total pregnancies [8]. Of note, in order to mitigate the declining fertility rates, China has implemented the two-child policy in 2016 and the third-child policy in 2021, respectively, leading to a dramatic increase in women with AMA and a better time window to investigate the outcomes of AMA in China [8]. Based on data published by the National Health and Family Planning Commission, 90 million couples are eligible for the two-child policy, among whom 60% of women were 35 and above years old, and 50% were above 40 years old [9]. Therefore, the increase of AMA in China is inevitable so that the investigation of outcomes of AMA is essential using the healthcare records collected in recent years, a period after the implementation of the two-child policy.

Although mounting evidence has revealed the adverse outcomes of AMA, including gestational diabetes mellitus (GDM), cesarean delivery, obesity, hypertension, hyperbilirubinemia, perinatal mortality, and maternal morbidity [10], there have been debates on the outcomes of AMA for years. According to a UK birth cohort study (2000–2002) [11], maternal age between 35 and 39 years was positively associated with cognitive ability, which showed the involvement of socioeconomically advantaged family background. In addition, mothers aged 35–39 years may present healthier behaviors during pregnancy, which is considered a benefit of AMA [11]. Given these contradictory results and the current trends in delayed fertility, the association between maternal age and pregnancy outcomes is an essential question to be addressed [6]. Thus, the objective of this study was to investigate whether adverse pregnancy outcomes varied by maternal age.

## Materials and methods

### Study design

We conducted a population-based retrospective study using the healthcare records data from the Medical Birth Registry in Xiamen (MBRX), China, between January 2011 and March 2018. A detailed description of the study methods has been published previously [12]. In brief, this is a registration system that was established in 2007 in Xiamen based on a compulsory notification of all live and stillbirths from the12th week of gestation onward. All women are registered at a community health center when they get pregnant, and then referred to a general hospital for health care from the 32nd week of gestation to delivery. Health checks are conducted at birth (within 3 days of birth) for all children, and annual examinations are conducted. Every woman and child are linked to individual records in the Xiamen Citizen Health Information System using the personal identification number. Every child is also linked to his/her biological mother’s ID number.

All women over 18 years of age who performed a 75 g oral glucose tolerance test (OGTT) between 24 and 28 weeks of gestation were eligible for inclusion. A total number of mother-child pairs in the healthcare records were 67,809 during the study period. After excluding the mother-child pairs lacking mother’s weight or height data (N = 179, 2.6%), missing children’s birth weight data (N = 3925, 5.8%), and multiple births (N = 568, 8.4%), a total of 63,137 mother–child pairs were included in our analysis. Information collected from MBRX on maternal factors contained maternal age, education, occupation, weight in 12 weeks before pregnancy (pre-pregnancy body mass index), numbers of pregnancy/infants, medical history, and clinical measurements including height, weight, blood pressure, fasting glucose, gestational diabetes screening results, and complications during pregnancy. Children’ healthcare records included information from newborns to preschool-aged children (date of birth, sex, gestational week of birth, Apgar score, weight, height, and so on). This study was approved by the ethics committee of the First Affiliated Hospital of Xiamen University (KYH2018–007) and conducted in accordance with the Declaration of Helsinki of 1975, revised in 2013.

### Variables definition

Large-for-gestational-age (LGA) was defined as a birth weight in the 90th percentile or above for gestational age[13]. Small-for-gestational-age (SGA) was defined as a birth weight less than the 10th percentile for gestational age. GDM was identified according to the reference values by International Association of Diabetes and Pregnancy Study Groups. Pregnant women were considered to have GDM if they met or exceeded any of the following plasma glucose values after undergoing an OGTT with 75 g glucose load between 24 and 28 weeks of gestation: fasting plasma glucose (FPG) ≥ 5.10 mmol/L, postload 1-hour glucose level ≥ 10.00 mmol/L and postload 2-hour glucose level ≥ 8.50 mmol/L [14]. Macrosomia was diagnosed with birth weight > 4,000 g. Low-birth weight (LBW) was defined as birth weight < 2,500 g. Preterm birth was defined as birth before 37 weeks of gestation.

### Statistical analysis

Data were presented as the mean ± standard deviation (SD) for continuous variables and number (n) and percentage (%) for categorical variables. The statistical significance of differences between different groups was assessed by Chi-square test for categorical variables and t test or Kruskal-Wallis test for continuous variables. The univariate and multivariate logistic regression analysis was used to evaluate the association between pregnancy outcomes and maternal age. Tests for trend were performed and P-trend values were derived. Confounding factors for adjustments included systolic blood pressure (SBP), pre-pregnancy body mass index (BMI), education, parity, GDM, infant sex, and birth weight. Statistical analysis was carried with SAS version 9.4. All tests were two-tailed and a p-value < 0.05 was considered statistically significant.

## Results

A total of 63,137 women who gave birth (live or stillbirth) in Xiamen between January 2011 and March 2018 were included, of which mothers aged 25–29 years accounted for the highest proportion (50.5%) and mothers aged < 25, 20–35, and ≥ 35 years for 15.6%, 25.1%, and 8.8%, respectively. Baseline maternal and infant characteristics of participants in each maternal age group were summarized in Table 1. The pre-pregnancy BMI, systolic blood pressure, diastolic blood pressure, and proportion of mothers with parity-time ≥ 2 all increased significantly with maternal age. Regarding the level of education, the group aged 20–29 years had highest percentage (80.3%) of pregnant women receiving ≥ 9 years of education, followed by the group aged 30–34 years (76.7%). According to results of OGTT conducted at 24–28 weeks, values of FPG, postload 1-hour glucose level, and postload 2-hour glucose level all increased with maternal age. For infant characteristics, the number of weeks when labor occurred was higher in group aged < 25 years (39.0 ± 3.8 weeks) and 20–35 years (39.0 ± 20.1 weeks) compared to group aged 30–34 years (38.7 ± 3.2 weeks) and ≥ 35 years (38.4 ± 1.5 weeks). The P-values for all of the above were less than 0.001.

**Table 1:** Maternal and infant characteristics by maternal age categories among study population (N = 63,137)

	Maternal age (years)				*P*-value
	< 25	25–29	30–34	≥ 35	
**Maternal characteristics**					
Number, n (%)	9872 (15.6)	31,864 (50.5)	15,834 (25.1)	5567 (8.8)	
Age, years	22.9±1.3	27.0±1.3	31.6±1.4	37.1±2.1	**< 0.001**
Pre-pregnancy BMI, kg/m^2^	20.4±2.7	20.7±2.8	21.6±2.9	22.4±2.9	**< 0.001**
SBP, mmHg	107.2±10.6	107.6±10.5	107.7±10.7	108.9±11.1	**< 0.001**
DBP, mmHg	64.8±7.7	65.5±7.8	65.8±8.0	66.5±8.3	**< 0.001**
Level of education, n (%)					
≤ 9 years	3206 (39.0)	5470 (19.7)	3192 (23.3)	1587 (32.2)	**< 0.001**
> 9 years	5008 (61.0)	22,304 (80.3)	10,523 (76.7)	3348 (67.8)	
Parity, n (%)					
1	5838 (64.4)	16,909 (55.9)	4194 (27.4)	634 (11.7)	**< 0.001**
≥ 2	3221 (35.6)	13,332 (44.1)	11,127 (72.6)	4795 (88.3)	
OGTT at 24–28 weeks					
Fasting plasma glucose, mmol/L	4.4±0.4	4.5±0.4	4.6±0.4	4.6±0.4	**< 0.001**
Postload 1-h, mmol/L	7.3±1.6	7.7±1.6	8.2±1.7	8.6±1.7	**< 0.001**
Postload 2-h, mmol/L	6.2±1.2	6.6±1.3	7.0±1.4	7.5±1.5	**< 0.001**
**Infant characteristics**					
Birth weight, kg	3.2±0.4	3.2±0.5	3.2±0.5	3.2±0.5	**< 0.001**
Infant sex, male, n (%)	2981 (30.2)	10,485 (32.9)	5017 (31.7)	1631 (29.3)	0.140
Gestational age at birth, weeks	39.0± 3.8	39.0±20.1	38.7±3.2	38.4±1.5	**< 0.001**

Data showed as Mean±SD and n (%). BMI, body mass index; SBP, systolic blood pressure; DBP, Diastolic blood pressure; OGTT, oral glucose tolerance test

As shown in Table 2, the incidence of GDM increased significantly with maternal age (P < 0.001), from 9.2% among mothers aged < 25 years to 35.1% among mothers aged ≥ 35 years. The proportion of pregnant women with hypertension, cesarean section mode, preterm birth, LGA, and macrosomia all increased significantly with maternal age (P < 0.001). In contrast, the frequencies of both SGA and stillbirth decreased with maternal age (P < 0.001). Moreover, the proportion of pregnant women delivering LBW infants was highest in group aged ≥ 35 years (6.0%), while the lowest proportion was observed in group aged 25–29 years (4.5%). For deliveries of infants with an Apgar score < 7 at 5 min, there was no significant differences among these groups (Table 2).

**Table 2:** Characteristics of pregnancy outcomes for different maternal age groups (N = 63,137)

	Maternal age (years)				*P-*value
	< 25	25–29	30–34	≥ 35	
**Pregnancy outcomes**					
GDM	911 (9.2)	4592 (14.4)	3642 (23.0)	1952 (35.1)	**< 0.001**
Hypertension	11 (0.1)	48 (0.2)	41 (0.3)	35 (0.6)	**< 0.001**
Cesarean	2301 (23.3)	9712 (30.5)	6754 (42.7)	3094 (55.6)	**< 0.001**
Preterm birth	428 (4.3)	1458 (4.6)	878 (5.5)	390 (7.0)	**< 0.001**
SGA	458 (4.6)	1420 (4.5)	617 (3.9)	203 (3.6)	**< 0.001**
LGA	1312 (13.3)	4390 (13.8)	3189 (20.1)	1275 (22.9)	**< 0.001**
Macrosomia	279 (2.8)	1014 (3.2)	559 (3.5)	216 (3.9)	**< 0.001**
LBW	454 (4.6)	1444 (4.5)	839 (5.3)	336 (6.0)	**< 0.001**
Stillbirth	413 (4.2)	1255 (3.9)	514 (3.2)	153 (2.7)	**< 0.001**
Apgar score < 7 at 5 min	14 (0.1)	27 (0.1)	23 (0.1)	2 (0.0)	0.057

Data showed as n (%). GDM, gestational diabetes mellitus; LBW, low birth weight; SGA, small-for-gestational-age; LGA, large-for-gestational-age

Table 3 presented univariate and multivariate logistic regression analysis of pregnancy outcomes by comparing different maternal age groups (< 25 years, 30–34 years, **≥** 35 years) to the reference group aged 25–29 years. After adjustment for SBP, pre-pregnancy BMI, education, infant sex, and parity, it was shown that mothers aged < 25 years were associated with lower risks of GDM (OR: 0.615, 95%CI: 0.529–0.715) compared to the reference group, while the mothers aged 30–34 years (OR:1.735, 95%CI: 1.586–1.899) and ≥ 35 years (OR: 2.705, 95%CI: 2.405–3.043) both exhibited substantially higher risks of GDM (P < 0.001). After further adjustment for GDM, it was shown that the risks of cesarean remained increased with age. Compared with women aged 25–29 years, AMA mothers **≥** 35 years were at higher risk of cesarean section (OR: 2.282, 95%CI: 2.057–2.533; P < 0.001), preterm birth (OR: 1.568, 95%CI: 1.279–1.922; P < 0.001) and delivering LBW infants (OR: 1.940, 95%CI: 1.566–2.404; P < 0.001). AMA mothers exhibited higher risk of hypertension (OR: 3.666, 95%CI: 1.923–6.988; P < 0.001) after accounting for GDM, pre-pregnancy BMI, education, infant sex, and parity. Statistical differences in risks for SGA, LGA, and stillbirth were observed among different age groups according to univariate logistic regression analysis, whereas these significant differences did not exist after multivariate logistic regression analysis (all P > 0.05). Of interest, the univariate analysis suggested AMA mothers were at a higher risk of macrosomia (OR: 1.228, 95%CI: 1.057 to 1.427), whereas, after adjustments of confounders including GDM, pre-pregnancy BMI, and infant sex, both AMA mothers and those aged 30–34 years were shown to be with lower risk of macrosomia, among whom AMA mothers were at the lowest risk (OR: 0.652, 95%CI: 0.494 to 0.859) compared to reference group. After further analysis, it was demonstrated that the infant sex was the crucial influencing factor contributing to the disparity, i.e., AMA mothers who gave birth to male babies had a lower risk of macrosomia (P = 0.045), while mothers who gave birth to female babies did not (P = 0.313). In addition, no significant difference was observed for Apgar score < 7 in different groups.

**Table 3:** Univariate and multivariate logistic regression analysis of pregnancy outcomes across different maternal age groups (N = 63,137)

Pregnancy outcomes		Maternal age (years)				*P trend* value
		< 25	25–29	30–34	≥ 35	
GDM*	Crude OR (95%CI)	0.604 (0.560 to 0.651)	Reference	1.774 (1.690 to 1.862)	3.207 (3.010 to 3.417)	< 0.001
	Adjusted OR (95%CI)	0.615 (0.529 to 0.715)	Reference	1.735 (1.586 to 1.899)	2.705 (2.405 to 3.043)	< 0.001
Hypertension†	Crude OR (95%CI)	0.739 (0.384 to 1.424)	Reference	1.721 (1.134 to 2.612)	4.194 (2.710 to 6.489)	< 0.001
	Adjusted OR (95%CI)	0.926 (0.399 to 2.150)	Reference	1.717 (0.975 to 3.022)	3.666 (1.923 to 6.988)	< 0.001
Cesarean‡	Crude OR (95%CI)	0.693 (0.658 to 0.731)	Reference	1.697 (1.631 to 1.765)	2.854 (2.693 to 3.024)	< 0.001
	Adjusted OR (95%CI)	0.692 (0.625 to 0.768)	Reference	1.550 (1.442 to 1.666)	2.282 (2.057 to 2.533)	< 0.001
Preterm birth‡	Crude OR (95%CI)	0.941 (0.842 to 1.050)	Reference	1.215 (1.115 to 1.324)	1.551 (1.382 to 1.741)	< 0.001
	Adjusted OR (95%CI)	1.017 (0.829 to 1.247)	Reference	1.170 (1.003 to 1.366)	1.568 (1.279 to 1.922)	< 0.001
LBW‡	Crude OR (95%CI)	1.016 (0.912 to 1.131)	Reference	1.179 (1.080 to 1.286)	1.354 (1.198 to 1.530)	< 0.001
	Adjusted OR (95%CI)	1.003 (0.813 to 1.237)	Reference	1.444 (1.233 to 1.692)	1.940 (1.566 to 2.404)	< 0.001
SGA‡	Crude OR (95%CI)	1.039 (0.932 to 1.157)	Reference	0.862 (0.783 to 0.950)	0.800 (0.689 to 0.930)	< 0.001
	Adjusted OR (95%CI)	1.058 (0.862 to 1.299)	Reference	1.070 (0.895 to 1.280)	1.082 (0.818 to 1.431)	0.850
LGA‡	Crude OR (95%CI)	0.833 (0.780 to 0.889)	Reference	1.367 (1.301 to 1.436)	1.601 (1.493 to 1.716)	< 0.001
	Adjusted OR (95%CI)	1.046 (0.923 to 1.185)	Reference	1.067 (0.975 to 1.169)	1.087 (0.958 to 1.233)	0.425
Macrosomia‡	Crude OR (95%CI)	0.885 (0.774 to 1.012)	Reference	1.113 (1.002 to 1.237)	1.228 (1.057 to 1.427)	0.001
	Adjusted OR (95%CI)	0.998 (0.779 to 1.277)	Reference	0.815 (0.677 to 0.981)	0.652 (0.494 to 0.859)	0.010
Stillbirth‡	Crude OR (95%CI)	1.060 (0.947 to 1.188)	Reference	0.812 (0.731 to 0.901)	0.680 (0.573 to 0.806)	< 0.001
	Adjusted OR (95%CI)	1.120 (0.903 to 1.390)	Reference	1.091 (0.900 to 1.323)	0.933 (0.676 to 1.287)	0.557
Apgar score < 7‡	Crude OR (95%CI)	1.675 (0.878 to 3.195)	Reference	1.715 (0.983 to 2.992)	0.424 (0.101 to 1.783)	0.071
	Adjusted OR (95%CI)	2.554 (0.786 to 8.298)	Reference	1.921 (0.644 to 5.729)	1.498 (0.285 to 7.889)	0.423

GDM, gestational diabetes mellitus; LBW, low birth weight; SGA, small-for-gestational-age; LGA, large-for-gestational-age; OR, odds ratio; CI, confidence interval. * was adjusted for systolic blood pressure, pre-pregnancy BMI, education, infant sex, and parity. † was adjusted for GDM, pre-pregnancy BMI, education, infant sex, and parity. ‡ was additionally adjusted for systolic blood pressure above †

## Discussion

In the present study, we aimed to investigate the association between adverse maternal and fetal outcomes and maternal ages in Xiamen, China. After adjustment for confounders such as pre-pregnancy BMI, education, infant sex, and parity, the multivariate logistic regression revealed that the risks of GDM and cesarean delivery both increased with maternal age. AMA mothers were at higher risks of hypertension, preterm birth, and LBW, whereas at lower risk of macrosomia when compared to mothers aged 25–29 years. In addition, infants delivered by AMA mothers did not exhibit an increased risk of low Apgar scores.

As one of the key observations in the present study, the risk of GDM increases simultaneously in association with maternal age, which is consistent with previous studies [4, 15, 16]. Mounting evidence has elucidated the potential mechanisms mediating AMA related to GDM including pancreatic β-cell dysfunction, deteriorated insulin sensitivity, and lipid metabolism disturbance, which may occur with maternal age [9, 17, 18]. In accordance with this, it was noted in the present study that the pre-pregnancy BMI also increased with maternal age. A study by Li et al. suggested that the higher risk of GDM in women with AMA may be due to a sedentary lifestyle and higher BMI [19]. It has been recommended that for AMA mothers, a healthy lifestyle may help to maintain a healthy internal environment, thus reducing the risk of maternal complications [9]. Therefore, for pregnant women with higher pre-pregnancy BMI, a strict weight control program and health education for mothers with AMA should be warranted.

In previous studies, Karlström and others found that the risk of cesarean delivery increased with maternal age, with the odds ratio 1.10 to 4.42 times for mothers aged > 35 years compared to their younger counterparts [20–23]. Similarly, we also found a significant association between cesarean delivery and elder maternal age in the multivariate analysis. According to several hypothetical reports focusing on the biological mechanisms underlying the high risk of cesarean delivery in AMA mothers, prolonged labor with age, slow progress of labor, impaired uterine contractility, and uterine dysplasia are the most common causes of cesarean delivery [24, 25]. Indeed, many of the cesarean deliveries were performed following the strong requests of AMA mothers. As demonstrated in a retrospective study conducted in Boston, women aged ≥ 40 years were more likely to choose cesarean section without any medical indication [26]. Dan et al. confirmed that selective cesarean section mode was elevated with increasing maternal age [9]. Therefore, the increased rates of cesarean delivery in AMA mothers might also be attributed to subjective choices made by AMA mothers, obstetricians, and gynecologists in order to avoid risks of adverse pregnancy and birth outcomes.

Although some studies showed no differences in birth weight among different ages [27, 28], our present study found that the higher risk of LBW was related to AMA. This finding was supported by several research indicating that elder mothers are more likely to have health issues, for instance, reduced cardiovascular reserve capacity, which can lead to poor placental perfusion and LBW [18, 29]. Additionally, we also observed that the rate of preterm birth was increased in the AMA age group, which is consistent with prior studies [21, 23, 30]. Of note, the association between AMA and LBW or preterm birth might be also affected by obstetrical history and socioeconomic factors [28, 31, 32]. In light of this, further adjustment for additional confounding factors, including previous adverse pregnancy outcomes, residence, insurance information, and smoking status will be required in future studies to validate this association based on the present population.

It is worthwhile to notice the relationship between AMA and the risk of macrosomia. Compared to women aged 25–29 years, AMA mothers were associated with an increased risk of macrosomia in the univariate analysis. In contrast, after adjustment for confounders including GDM, pre-pregnancy BMI, infant sex, etc., the trend in risk of macrosomia was reversed, with AMA mothers carrying the lowest risk of macrosomia (OR: 0.652, 95% CI: 0.494 to 0.859, P = 0.010). Further analysis revealed that infant sex was the key influencing factor leading to the discrepancy, in which circumstance, a lower risk of macrosomia (P = 0.045) was observed in AMA mothers delivering male infants, but not those delivering female infants (P = 0.313). In contrast to our result, studies by Stotland et al. and others demonstrated a higher risk of macrosomia for male infants [33–35]. Therefore, further studies are needed to elucidate the underlying mechanisms regarding sex-specific differences in macrosomia. Moreover, in the univariate analysis, we observed that AMA was associated with higher risks of SGA, LGA, and stillbirth (all P < 0.001). Nonetheless, after adjustment for multiple confounders such as SBP, pre-pregnancy BMI, education, child sex, and parity, the associations were significantly attenuated (all P > 0.05). Thus, we speculate that the confounders of maternal characteristics and pregnancy complications played an essential role. Additionally, consistent with Dulitzki et al. [19, 36], our study revealed that the risk of 5-minute Apgar scores < 7 was not affected by maternal age.

Although our study has included large sample size and conducted multiple adjustments of confounders, there are several limitations that should be noted. Firstly, this is an observational study so we cannot exclude the presence of potential unmeasured or residual confounding, as well as overadjustment bias. The lack of available gestational weight gain (GWG) data and previous adverse pregnancy outcomes information in our present study, at least in part, may affect the result. In addition, we cannot distinguish elective and emergency cesarean deliveries according to the present healthcare records, so we could not determine the association between AMA and the risk of emergency cesarean section. It is noteworthy that the hypertension was diagnosed based on the blood pressure values extracted from the database. Thus, the lack of information on the medication for hypertension may lead to missed diagnoses. Besides, previous large population-based study [37] had revealed that advanced maternal age is significantly associated with an increased risk of hypertensive disorders of pregnancy such as preeclampsia. However, due to the lack of data on pregnancy related hypertensive disorders, it is difficult for us to apply further analysis. Finally, this study was performed based on the population from Xiamen, China, which may limit the generalization of the findings in other countries or ethnicities.

## Conclusion

AMA was associated with several adverse perinatal outcomes including GDM, hypertension, cesarean, preterm birth, and LBW, even after adjustment for confounders. Given the continuing rapid increase of maternal age in China, healthcare providers should be aware of these maternal and fetal risks in order to offer professional suggestions and better health cares for AMA mothers during pregnancy.

## Data Availability

The data used during the current study are available from the corresponding author on reasonable request.

## Abbreviations

AMA: Advanced maternal age
GDM: Gestational diabetes mellitus
LBW: Low-birth weight, LGA:large-for-gestational-age
MBRX: Medical Birth Registry in Xiamen
OGTT: Oral glucose tolerance test
SGA: Small-for-gestational-age
FPG: Fasting plasma glucose
DBP: Diastolic blood pressure
BMI: Body mass index
SD: Standard deviation
GWG: Gestational weight gain
